# Study of MC:DN-Based Biopolymer Blend Electrolytes with Inserted Zn-Metal Complex for Energy Storage Devices with Improved Electrochemical Performance

**DOI:** 10.3390/membranes12080769

**Published:** 2022-08-08

**Authors:** Elham M. A. Dannoun, Shujahadeen B. Aziz, Rebar T. Abdulwahid, Sameerah I. Al-Saeedi, Muaffaq M. Nofal, Niyaz M. Sadiq, Jihad M. Hadi

**Affiliations:** 1 Associate Chair of the Department of Mathematics and Science, Woman Campus, Prince Sultan University, P.O. Box 66833, Riyadh 11586, Saudi Arabia; 2 Hameed Majid Advanced Polymeric Materials Research Laboratory, Physics Department, College of Science, University of Sulaimani, Qlyasan Street, Kurdistan Regional Government, Sulaimani 46001, Iraq; 3 The Development Center for Research and Training (DCRT), University of Human Development, Kurdistan Region, Sulaymaniyah 46001, Iraq; 4 Department of Physics, College of Education, University of Sulaimani, Old Campus, Sulaimani 46001, Iraq; 5 Department of Chemistry, College of Science, Princess Nourah bint Abdulrahman University, P.O. Box 84428, Riyadh 11671, Saudi Arabia; 6 Department of Mathematics and Science, Prince Sultan University, P.O. Box 66833, Riyadh 11586, Saudi Arabia; 7 Department of Medical Laboratory of Science, College of Health Sciences, University of Human Development, Kurdistan Regional Government, Sulaimani 46001, Iraq

**Keywords:** plasticized polymer composite, impedance spectroscopy, electrical equivalent circuit design, cyclic voltammetry, transference number measurement and linear sweep voltammetry, electrical double-layer capacitor device

## Abstract

Stable and ionic conducting electrolytes are needed to make supercapacitors more feasible, because liquid electrolytes have leakage problems and easily undergo solvent evaporation. Polymer-based electrolytes meet the criteria, yet they lack good efficiency due to limited segmental motion. Since metal complexes have crosslinking centers that can be coordinated with the polymer segments, they are regarded as an adequate method to improve the performance of the polymer-based electrolytes. To prepare plasticized proton conducting polymer composite (PPC), a simple and successful process was used. Using a solution casting process, methylcellulose and dextran were blended and impregnated with ammonium thiocyanate and zinc metal complex. A range of electrochemical techniques were used to analyze the PPC, including transference number measurement (TNM), linear sweep voltammetry (LSV), cyclic voltammetry (CV), galvanostatic charge–discharge (GCD), and electrochemical impedance spectroscopy (EIS). The ionic conductivity of the prepared system was found to be 3.59 × 10^−3^ S/cm using the EIS method. The use of glycerol plasticizer improves the transport characteristics, according to the findings. The carrier species is found to have ionic mobility of 5.77 × 10^−5^ cm^2^ V^−1^ s^−1^ and diffusion coefficient of 1.48 × 10^−6^ cm^2^ s^−1^ for the carrier density 3.4 × 10^20^ cm^−^^3^. The TNM revealed that anions and cations were the predominant carriers in electrolyte systems, with an ionic transference value of 0.972. The LSV approach demonstrated that, up to 2.05 V, the film was stable, which is sufficient for energy device applications. The prepared PPC was used to create an electrical double-layer capacitor (EDLC) device. The CV plot exhibited the absence of Faradaic peaks in the CV plot, making it practically have a rectangular form. Using the GCD experiment, the EDLC exhibited low equivalence series resistance of only 65 Ω at the first cycle. The average energy density, power density, and specific capacitance values were determined to be 15 Wh/kg, 350 W/kg, and 128 F/g, respectively.

## 1. Introduction

Polymers offer a broader range of applications than other materials, and their industry has grown at a faster rate. Solid polymer electrolyte (SPE) is one of their applications that has received a lot of attention. Inorganic salts are dissolved in a polymer matrix that contains heteroatoms such as O, N, and S in order to produce SPE [1,2,3]. Biopolymers are abundant in nature, affordable in cost, and have excellent solvent compatibility and film formation stability [4,5]. In polymer electrolytes, researchers frequently used biopolymers such as cellulose, starch, and carrageenan as polymer hosts [6,7,8].

Combining two or more natural polymers to create an eco-friendly host polymer is among the common methods to achieve good ionic conductivity, outstanding thermal characteristics, robust mechanical properties, and non-toxicity [9,10]. Cellulose is nature’s most abundant organic polymer, which makes it an excellent renewable resource [11]. Moreover, cellulose has the potential to replace petrochemical polymers as an alternative organic material [12]. Methyl cellulose (MC) is widely available, inexpensive, and beneficial to the environment. Methylation of alkali cellulose (MC) yields biodegradable polymer with excellent film-forming capabilities, the capacity to make transparent films, and exceptional mechanical and electrical characteristics [13,14]. Many qualities of the MC make it particularly suitable for this purpose. These include its outstanding film-forming and solubility capabilities and its excellent mechanical/thermal/amorphous/chemical stabilities [15,16]. Dextran (DN) is a natural polymer generated by Leuconostoc mesenteroides fermentation [17]. In the medical field, DN is often employed as a medication carrier [18]. The polymer chain of DN is made up of 1,6-D-glucopyranosidic links, with distinct oxygen-containing functional groups in this structure being the most advantageous for ionic conduction [19]. A polymer electrolyte is produced with these outstanding characteristics [20].

Many energy storage systems, such as supercapacitors (SCs) or batteries, are now utilizing polymer electrolytes as an electrolyte material. Charge storage in an electrical double-layer capacitor (EDLC) is established through production of Helmholtz double layers by ion mobility within the electrolyte in the absence of any electrode–electrolyte contact [21]. Owing to remarkable properties such as eco-friendliness, high cyclability, durability, safety, and high power density, EDLCs have gained popularity and are now considered much more desirable than batteries [22]. Furthermore, the device’s storage system allows it to be charged and discharged swiftly in a matter of seconds utilizing EDLCs [23]. Due to its high microporosity, activated carbon (AC) has been commonly used in commercial EDLCs because it provides high capacitance values [24].

Metal complexes have been found to be important in enhancing the amorphous phase of polar polymers in prior research [25]. Increasing the amorphous phase has the potential to improve DC ionic conductivity [26]. According to Brza et al. [27,28], adding metal complex to a polyvinyl alcohol (PVA) host caused an increment in the amorphous regions and thereby higher EDLC device performance. Due to the increased amorphous phase, metal complexes boost the ion transport parameters such as mobility (*µ*) of ions, their diffusion coefficient (D), and also charge carrier density (*n*) [25,27,28]. Therefore, inserting a zinc (Zn) (II) metal complex in a chitosan-based electrolyte has the primary goal of improving the amorphous phase for ion conduction. Salts were chosen because of their small cation size, large anion size, and low lattice energy. Ammonium salts have been shown to have a strong ionic conductivity and good thermal stability. The polymer electrolyte local viscosity is reduced by introducing salt and employing glycerol as a plasticizer, resulting in increased ion mobility and improved ion conductivity [29,30,31]. Glycerol is used as a plasticizer to improve conductivity because of the existence of many hydroxyl groups (OH) in its structure. There is a good compatibility between glycerol and commonly used biopolymers. Andrade and colleagues [32] improved the conductivity to 4.7 × 10^−4^ S/cm of a pectin–lithium perchlorate (PN-LiClO_4_) electrolyte by inserting glycerol. When it comes to EDLCs, the electrolyte is the most important component since it ensures the charge movement between the two electrodes. Liquid electrolytes, in general, have a number of practical drawbacks, including leakage, chemical hazards, thermal volatility, safety, and electrode corrosion. To overcome these limitations, various studies have been carried out to substitute the liquid electrolyte with a safer and stable electrolyte, such as polymer-based electrolytes, for energy device applications. Thus, in the current study, EDLCs are fabricated with polymer-based electrolyte as a mediator between the two electrodes. Based on our previous reports [28,33] on metal complexes and their roles in amorphous phase improvement, Zn-metal complex is added to MC:DN polymer blends in the current work. In our previous work [31], we observed that MC:DN:ammonium thiocyanate (NH_4_SCN):glycerol is an efficient electrolyte for EDLC applications. The result of the present work reveals that Zn-metal complex improves some parameters associated with EDLC devices.

## 2. Experimental Methods

### 2.1. Materials and Electrolyte Preparation

The raw ingredients used in this work were methyl cellulose (MC) (viscosity: 4000 cP) and dextran (DN) (molecular weight (Mw): 35,000–45,000) powders, which were obtained from Sigma-Aldrich (Kuala Lumpur, Malaysia). In order to create the MC:DN blended polymer, two beakers with 50 mL of distilled water were used to dissolve 70 wt.% MC and 30 wt.% DN separately at room temperature under continuous stirring with a magnetic stirrer for three hours. The achieved solutions were then combined and agitated for four hours to achieve a homogeneous blended solution. The MC:DN:NH_4_SCN electrolyte was then created by adding a set amount of NH_4_SCN (40 wt.%) to the blended MC:DN solution while stirring continuously for one hour. Plasticization was achieved by adding 42 wt.% glycerol to this homogeneous dispersed mixture and stirring continuously for one hour until a clear solution was formed. After that, prepared diluted Zn-metal complex (10 mL) was added to the plasticized sample of MC:DN:NH_4_SCN:glycerol for two hours.

In previous work, the methodology for preparing metal complexes as a green method was documented [33]. For the last film preparation step, the mixture was slowly poured into a dry clean Petri dish. The casted film had two weeks to reach complete dryness at room temperature. Transferring the film to a desiccator for another week provided additional drying. As a result, the technique produced film that was solvent-free. Finally, the obtained free-standing film was peeled off the Petri dish for further characterizations and EDLC device fabrication.

### 2.2. EIS Analysis

The impedance trends of the prepared polymer electrolytes at room temperature were examined using electrochemical impedance spectroscopy (EIS) with model HIOKI 3532-50 LCR HiTESTER (Nagano, Japan) (50 Hz ≤ *f* ≤ 1000 kHz). The electrolytes were packed in between a couple of electrodes (stainless steel) in order to achieve this result. The information gathered from this investigation was utilized to investigate the electrolytes’ ionic conductivity and dielectric properties.

### 2.3. TNM and LSV Measurements

A V&A Instrument (Neware) (V & A Instrument, Shanghai, China) DP3003 digital DC power supply was used to record the transference number (TNM) through the DC polarization technique. Briefly, 0.8 V was used as a constant potential to polarize the two electrodes (held in Teflon cases), that sandwiched the prepared electrolyte to record the DC current response over time, at ambient temperature.

In this study, the potential stability of the prepared ion-conducting polymer electrolyte was assessed by a Neware DY2300 potentiostat (Digi-Ivy, Neware, Shenzhen, China) using the linear sweep voltammetry (LSV) technique at a 10 mV/s scan rate.

### 2.4. EDLC Preparation

Carbon black, activated carbon (AC), and polyvinylidene fluoride (PVdF) were used during the synthesis of the electrode. The preparation of electrodes was presented thoroughly in a prior work [34]. The electrode thickness was set at 25 mm. The maximum conductive electrolyte was then located between two carbon electrodes in a coin cell type CR2032. The cyclic voltammetry (*CV*) in the potential range of 0.0 to 0.9 V for the fabricated EDLC was acquired using a Digi-IVY DY2300 Potentiostat (Digi-Ivy, Neware, Shenzhen, China) at varied sweep rates (10 to 100 mV/s). From the *CV* curve, the specific capacitance (*C_s_*) was extracted using Equation (1):

(1) $$C_{CV}=\int_{V_{i}}^{V_{f}}\frac{I\left(V\right)dV}{2mv\left(V_{f}-V_{i}\right)}.$$

*I(V)dV* (the area of the *CV* curve) is defined as the mass of the active material and the rate at which it is scanned. According to this research, *V_f_* is 0.9 V, whereas *V_i_* starts from 0 V. The charge–discharge characteristics of the designed EDLC at a current density of 0.5 mA/cm^2^ were investigated using a Neware battery cycler. Equations (2)–(5) were used to subtract the central parameters related to the EDLC device as follows:

(2) $$C_{CD}=\frac{i}{sm},$$

(3) $$ESR=\frac{V_{d}}{i_{}},$$

(4) $$E=\frac{C_{s}V^{2}}{2},$$

(5) $$P_{}=\frac{V^{2}}{4m(ESR)}.$$

To signify the applied current and discharge area gradient, the letters *i* and *s* are used; for the applied potential and potential drop, *V* and *V_d_* are used.

## 3. Results and Discussion

### 3.1. Impedance Study

**Figure 1:**
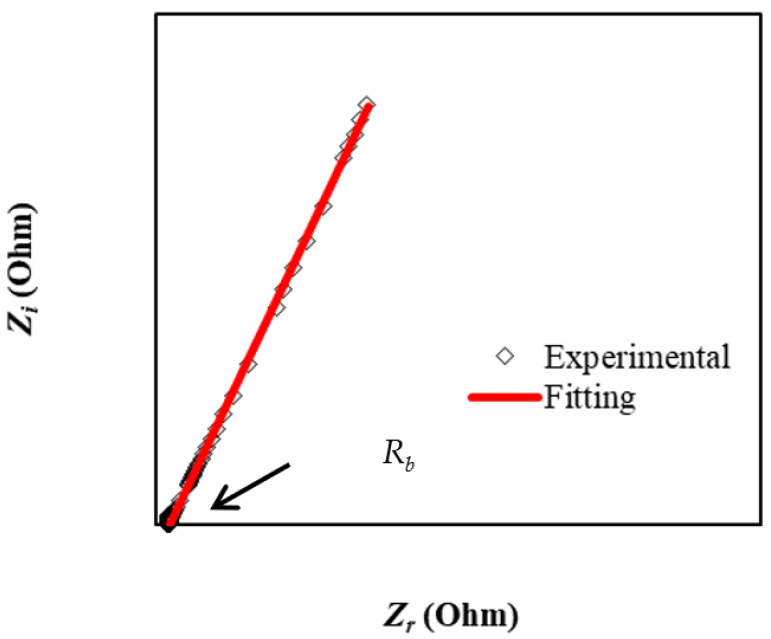
The impedance plot of glycerolized MC:DN:NH_4_SCN:Zn (II) metal complex electrolyte films at ambient temperature.

Impedance spectroscopy (IS) is a useful tool for determining the ionic conductivity of polymeric materials, as well as for determining the electrical characteristics of novel materials that will be employed in electrochemical devices [35,36]. Due to their broad range of applications in solid electrochemical devices, ion-conducting materials have received much interest in recent years [37]. Commonly, the impedance plot of polymer electrolyte displays a semicircle in the high-frequency area in addition to a tail in the low-frequency region [38]. Figure 1 shows the impedance pattern of glycerolized MC:DN:NH_4_SCN:Zn (II) metal complex electrolyte films at ambient temperature. Ionic conduction is characterized by a compressed semicircle at high frequency, for example, charge transfer resistance (CTR) in bulk material and a low-frequency tail owing to the electrode polarization influence are often distinguished by IS [39,40,41]. Composite samples’ impedance graphs using the electrical equivalent circuit (EEC) model are shown in Figure 1. Impedance plots from the experiment were compared to the EEC model to provide a clear image of the systems being studied. In the insets of Figure 1, we can see the analogous circuits. Low-frequency data from Figure 1 show the polarization effect that occurs due to the electric double layer formed at the interface of the electrode and electrolyte. Electrolyte/electrode interface capacitance may be calculated using blocking electrodes in the impedance analysis. In order to achieve optimum capacitance, the impedance graph shown above should display a vertical spike in the low-frequency range. As a result, instead of a vertical spike, we saw one had an angle of less than 90 degrees. It is possible that the tilt in the spike resulted from the electrodes forming a double layer and the electrolyte or electrodes’ surface being rough [42,43].

The impedance was divided into two parts by software: one for the real (*Z_r_*) and one for the imaginary (*Z_i_*). From the *Z_r_* and *Z_i_* data given as a Nyquist plot, the bulk resistance (*R_b_*) was determined using the intersection with the real impedance axis (*Z_r_*). Conductivity may be calculated on the basis of the formula shown below [44]:

(6) $$\sigma_{dc}=\left[\frac{1}{R_{b}}\right]\times \left[\frac{t}{A}\right].$$

The thickness of the film is given by *t*, and its area is given by *A* in Equation (6). Different EEC parameters are presented in Table 1 including constant phase elements (*CPE*) for the electrolyte. The charge resistance is used to determine the DC conductivity, which is listed in Table 2 with other ion transport parameters. The conductivity is 3.59 × 10^−3^ S/cm in the present work which is higher than that reported previously (3.08 × 10^−4^ S/cm) [31] for chitosan (CS):DN:NH_4_SCN:gly without metal complex.

Figure 1 indicates that the plasticizer is responsible for the absence of semicircles in the high-frequency zone. Equation (7) can be employed to estimate the value of *CPE* impedance (*Z_CPE_*) [45]:

(7) $$Z_{CPE}=\frac{1}{C\omega^{p}}\left[\cos\left(\frac{\pi p}{2}\right)-i\sin\left(\frac{\pi p}{2}\right)\right].$$

The capacitance of the *CPE* element, angular frequency, and deviance of the plot from the real axis are represented by *C*, ω, and *p*, respectively. The *CPE* is linked in series with the electrolytes’ *R_b_*, and their EIS signals only show the spike. Equations (8) and (9) can be used to determine the impedance numerically [45]:

(8) $$Z_{r}=\frac{\cos\left(\frac{\pi p}{2}\right)}{C\omega^{p}}+R_{b},$$

(9) $$Z_{i}=\frac{\sin\left(\frac{\pi p}{2}\right)}{C\omega^{p}}.$$

In order to obtain the *R_b_* values precisely, in the fitting parameters (*CPE*1 and *CPE*2), these equations are also used. Increased ion numbers in the electrolytes can explain the high *CPE* results [46].

Generally, pure polymers such as MC and DN, which do not contain mobile carriers, have weak room temperature ionic conductivity, whereas the addition of dopant salt, plasticizer, and metal complex plays a major role in elevating the conductivity of the host medium. This enhancement in ionic conductivity is caused by a couple of important factors. Initially, the addition of dopant salt and its dissociation rate of salt to its constituents result in an increase in the number of available free ion species within the host medium for conduction, which obviously enhances ionic conductivity. Secondly, both the plasticizer and metal complex have a considerable impact on the structural characteristics and formation of interconnecting amorphous domains of the polymer blend. This enhances chain rotational freedom and segmental motion, allowing for greater ion transport and minimizing the ion cloud retardation effect, which eventually leads to a better ionic conductivity.

As the impedance data consist only of a single spike, the diffusion coefficient (*D*) can be calculated from the following equation [27,34]:

(10) $$D=D_{{}^{\circ}}\exp\{-0.0297{\left[\ln D_{{}^{\circ}}\right]}^{2}-1.4348\ln D_{{}^{\circ}}-14.504\},$$

where

(11) $$D_{{}^{\circ}}=\left(\frac{4k^{2}l^{2}}{R_{b}{}^{4}\omega_{\min}{}^{3}}\right),$$

where *ω*_min_ is angular frequency related to the smallest *Z_i_* value and the thickness of the sample is denoted by *l*. For the current electrolyte system, the ion transport parameters are tabulated in Table 2.

The Nernst–Einstein relation can be used to estimate the ionic mobility (*µ*) as presented in Equation (12).

(12) $$\mu =\left(\frac{eD}{K_{b}T}\right)$$

where *T* and *k_b_* have normal meanings.

Equation (13) shows the calculation for ionic conductivity ($\sigma_{DC}$):

(13) $$\sigma_{DC}=ne\mu .$$

Consequently, the density of charge carrier (*n*) can be determined from the following equation:

(14) $$n=\left(\frac{\sigma_{DC}K_{b}T\tau_{2}}{{\left(eK_{2}\varepsilon_{o}\varepsilon_{r}A\right)}^{2}}\right).$$

In Table 2, the transport parameters are presented.

### 3.2. TNM Analysis

**Figure 2:**
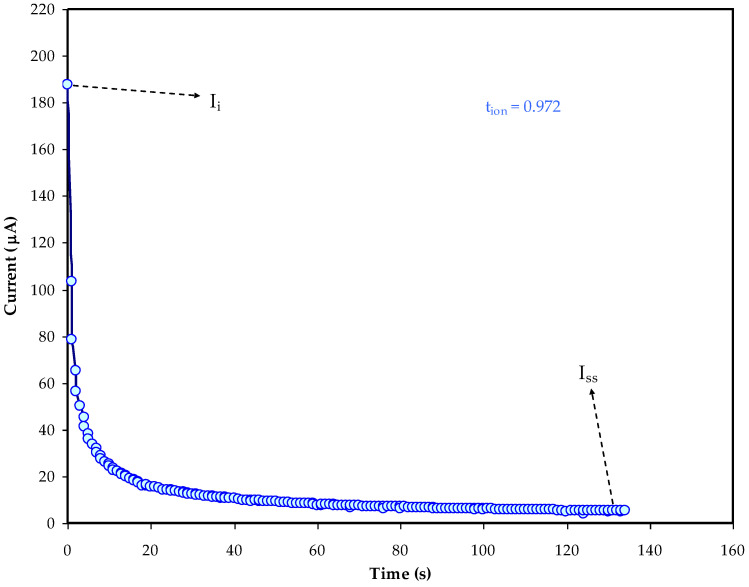
The trend of current over time throughout polarization for the MC:DN:NH_4_SCN:Gly:Zn electrolytes.

To establish the major charge carrier species within the electrolytes, the transference number measurement (TNM) is used. The dominancy of ions within a polymer electrolyte can be confirmed once its value approaches unity [47]. The current as a function of time during polarization for the MC:DN:NH_4_SCN:Gly:Zn electrolyte is displayed in Figure 2. From the figure, it was observed that the initial current for the system decreases over time, since the electrolyte is depleted of ionic species [48,49]. At the beginning of the polarization, a high current value of above 180 µA may be observed, suggesting that electrons and ions have been carried. As ions from the electrolyte are trapped on the stainless steel electrodes, the current decreases substantially as time passes and reaches 10 s. The plasticized electrolyte system’s increased electron mobility and decreased charge carriers are the primary causes of the initial current drop. This will ultimately reach a steady state when the diffusion process balances the movement of mobile ions [48,49]. The electrodes with stainless steel material act as a barrier for ion transport and only electrons contribute to current generation within the process of polarization [50]. To calculate the values of the *t_e_* and *t_ion_* for the electrolyte system, the following relationships were used:

(15) $$t_{ion}=\frac{I_{i}-I_{SS}}{I_{i}},$$

(16) $$t_{ion}=1-t_{el}.$$

The ionic transference is symbolized by *t_ion_*, the electron transference is symbolized by *t_el_*, the initial current is indicated by *I_i_*, and the steady-state current is designated by *I_ss_*. The calculated *t_e_* and *t_ion_* are 0.028 and 0.972, correspondingly, using the preceding equations.

### 3.3. LSV Analysis

**Figure 3:**
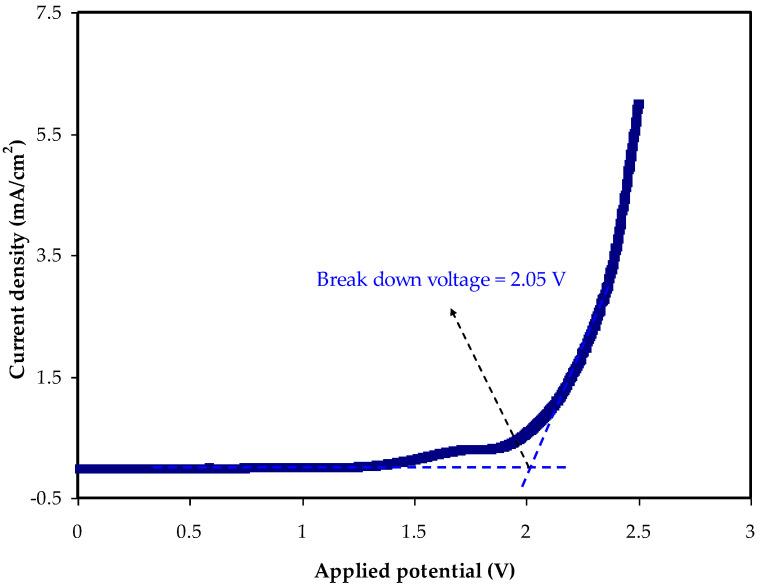
LSV plot for the MC:DN:NH_4_SCN:Gly:Zn electrolyte.

The LSV technique was used to measure the breakdown potential of the glycerolized MC:DN:NH_4_SCN:Zn (II) metal complex. Before using electrolytes in energy devices such as solar cells, batteries, and SCs, it is crucial to understand at what potential the electrolyte will begin to oxidize and degrade [51]. This is to guarantee that if different electrode materials are used, the electrolyte can sustain the voltage delivered and does not decompose. As seen in Figure 3, the electrolyte is steady, since no current fluctuates below the potential of 2.05 V. This implies that the prepared electrolyte has a wide potential window from 0 to 2.05 V. Beyond 2.05 V, as a result of the breakdown of the electrolyte at the inert electrodes, a large current is generated. This is owing to polymer collapse caused by disruption of polymer–polymer and polymer–salt interactions [52]. The plasticized system can show an enhancement in its electrochemical potential window upon the insertion of Zn-metal complex. Nonetheless, a satisfactory high breakdown voltage for both electrolyte systems was recorded in this research that best fit the EDLC application, which only required a potential window of 1.0 V [53].

### 3.4. CV Analysis

**Figure 4:**
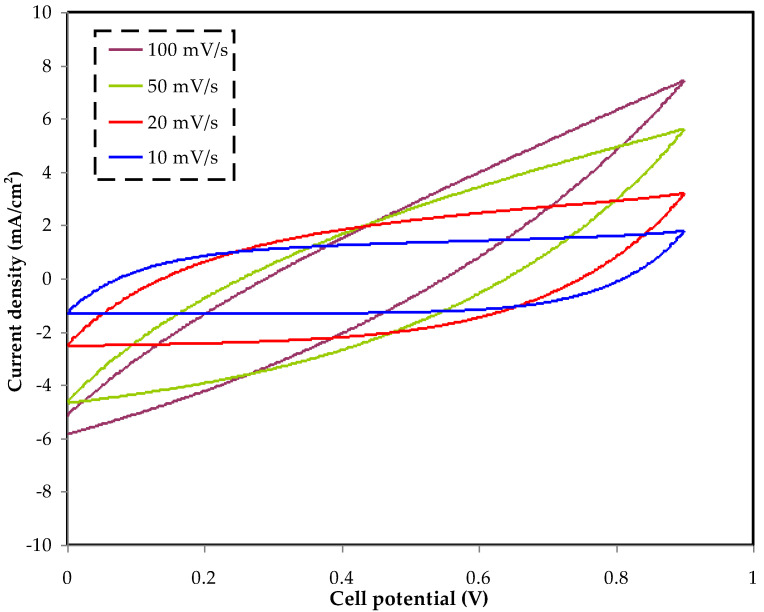
The CV profile for glycerolized the MC:DN:NH_4_SCN:Zn (II) metal complex electrolyte.

CV analysis is used to determine how an EDLC’s electrode–electrolyte interfaces store charge. For the electrolyte, the CV is performed at various scan rates, as shown in Figure 4. With reducing scan rate, the curves changed from a leaf-like shape to a more rectangular shape, as shown in Figure 4. The slight change from a perfect rectangular form can be caused by the internal resistance and the porosity of the used electrodes. The leaf-like shape of the CV profile is caused by the porosity of the AC electrodes, which is caused by a considerably large internal resistance [54]. The CV curves of the manufactured EDLC employing glycerolized the MC:DN:NH_4_SCN:Zn (II) metal complex electrolyte show no peaks, indicating that there is no oxidation or reduction in the EDLC. This shows that an electrical double-layer capacitor exists. The lack of redox peaks shows that there has not been a quick, reversible Faradaic reaction along with the formation of a double layer, which supports its EDLC characteristics. This implies that the charge–storage process in an EDLC is based on the fact that ions gather at the interfaces between the electrodes and the electrolyte when an electric potential is applied. As the process of charging starts, the induced electric field makes the EDLC electrodes exert force on free cations and anions within the electrolyte in a way that cations approach the negative electrode, while anions go to the positive electrode. The ions and electrons from the electrolyte and electrode, respectively, will be held in place by the electrostatic force created by the strong electric field, forming an electric charge double layer [55,56]. In this approach, an electric charge double layer forms on the carbon electrode surface, in which the energy is stored as potential energy [57]. Using Equation (1), the magnitudes of *C_s_* for the prepared system at various scan speeds are computed and presented in Table 3. When the scan rate is raised, the *C_s_* values are seen to drop. To put it another way, this is because as the scan rate increases, the quantity of stored charges on the electrode surface diminishes, resulting in more energy losses and lower Cs values [58].

### 3.5. GCD Study

**Figure 5:**
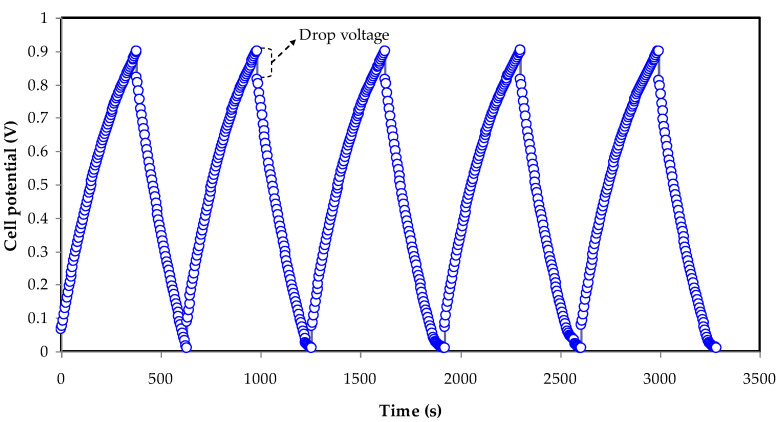
The GCD performances of the fabricated EDLC.

**Figure 6:**
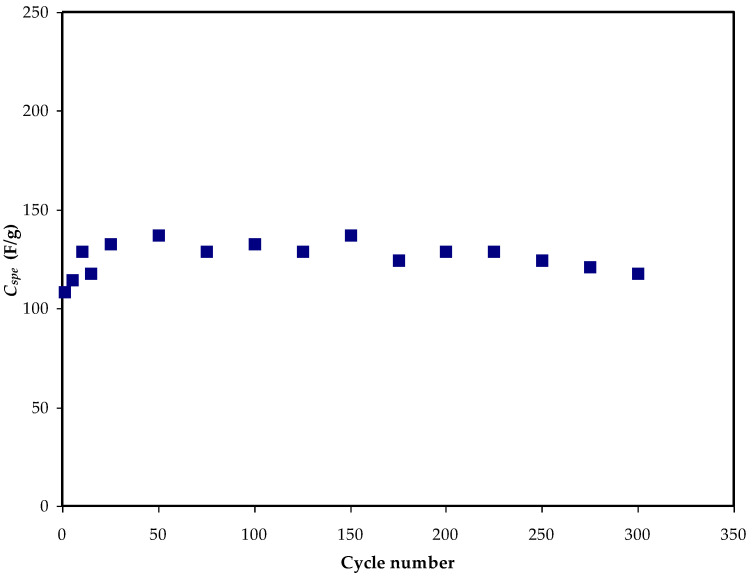
EDLC specific capacitance outcome over 300 cycles.

The profile of constructed EDLCs means galvanostatic charge–discharge (GCD) (Figure 5) can explain the EDLC’s cyclic durability in addition to the charging and discharging operations that occur in the system [59]. The ions move from the bulk electrolyte to the interfacial area when the current is introduced to the system, resulting in a charge double layer. Discharge patterns show rapid voltage drops, which are caused by ohmic losses all over the equivalent series resistance (*ESR*) of the cell. The voltage drop of discharge characteristics moderately deviates from the perfect triangle-like shape, which may be attributed to the internal resistance; additionally, both the roughness of the activated carbon and bulk electrolytes have an effect. The capacitive performance of the EDLC can be confirmed using the GCD profile, which has a nearly linear discharge slope [60]. The polarization process is indicated by the linearity of both charging and discharging processes, while conventional batteries are non-linear [61]. The *C_s_* from the charge–discharge curves can be computed using Equation (2). Figure 6 shows the determined *C_s_* magnitudes over the 300 cycles. The *C_s_* at the initial cycle is determined to be ~105 F/g that is equivalent to the *C_s_* value attained via CV analysis, proving that the EDLC displayed capacitor cell properties [62]. Then, at the 50th cycle, the *C_s_* was raised to ~135 F/g, and it remained nearly constant at ~128 F/g until the 300th cycle. In comparison to the *C_s_* addressed by Aziz et al. [60], the *C_s_* obtained in this study is higher for manufactured EDLCs, which is ~76.7 F/g for 100 cycles. As a result, the investigated electrolyte in this study can be used to fabricate EDLCs with high specific capacitance as a novel material. Hadi and coworkers [45] also documented the average *C_s_* value of ~19.5 F/g for their electrolyte system containing a CS–ammonium iodide(NH_4_I)–glycerol–Zn-metal complex. Previously published work based on MC:DN:NH_4_SCN has reported a 133 F g^−1^ system at room temperature [31].

**Figure 7:**
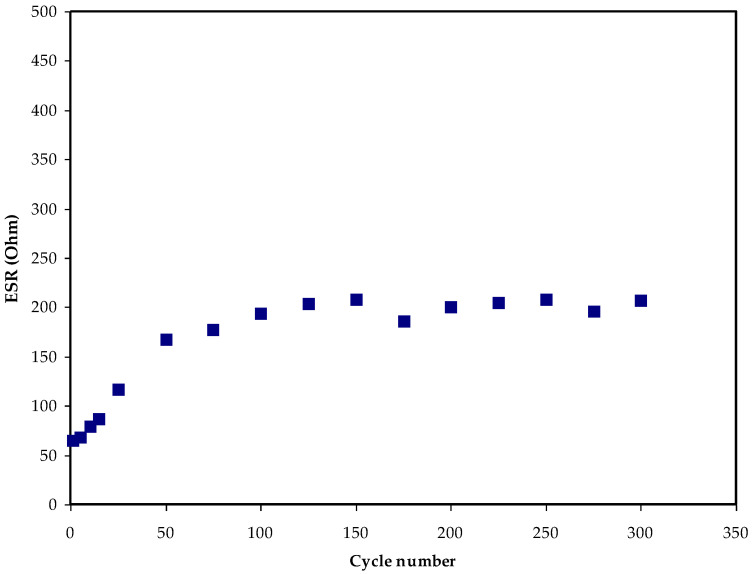
The equivalence series resistance *ESR* pattern for the fabricated EDLC.

Capacitance can be referred to as the ratio of electrical charge variation with respect to the potential changes for a particular system. For an EDLC system, there is generally a small potential fall during the discharge process, which is caused by its internal resistance. Figure 7 illustrates the fluctuation of the manufactured EDLC’s equivalent series resistance (*ESR*) versus cycle number. Equation (3) was used to calculate the EDLC *ESR* value [63]. At less than 200 cycles, the voltage loss is about 0.14 V. Since the process of polarization is impeded by the increase in *ESR*, voltage drop began to increase after 200 cycles, as illustrated in Figure 7. During a quick charge–discharge process, certain ions recombine to produce neutral ion pairs when the cycle number is high. Ion pairs can cause incorrect polarization to form on the electrodes’ surfaces [64]. On the other hand, between the 200th to 300th cycles, voltage fell and *ESR* began to stabilize. Cations and anions established a steady double layer process at some point during charge–discharge, resulting in stable internal resistance.

In a study by Arof et al. [65], it was revealed that each of the aluminum foils used as current collectors, the cycling process of the electrolyte, and electrode–electrolyte contact gap are the main contributors to establishing the internal resistance within the EDLC. The low *ESR* value shows good electrode-to-electrolyte contact and shows that ions move easily toward the electrode surface to form an electrical double layer [66]. The CS–MC–NH_4_SCN combination shows a similar trend, with the values of *ESR* slightly rising and *C_s_* values remaining constant [67]. Furthermore, rapid charging and discharging cause free ions to recombine, resulting in the formation of an ion pair, which reduces conductivity.

For the CS:MC:NH_4_I system, the *ESR* trend was the same [68]. The *ESR* value of a CS:starch:NH_4_I system was much higher than that of the present work [69]. In a prior work, the average *ESR* value in a system composed of a PVA:NH_4_SCN:Cd(II) complex was 51 Ω [70].

The EDLC’s performance is also described by energy density (*E_d_*) and power density (*P_d_*), which are two decisive parameters. Equations (4) and (5) can be used to approximately calculate these parameters.

**Figure 8:**
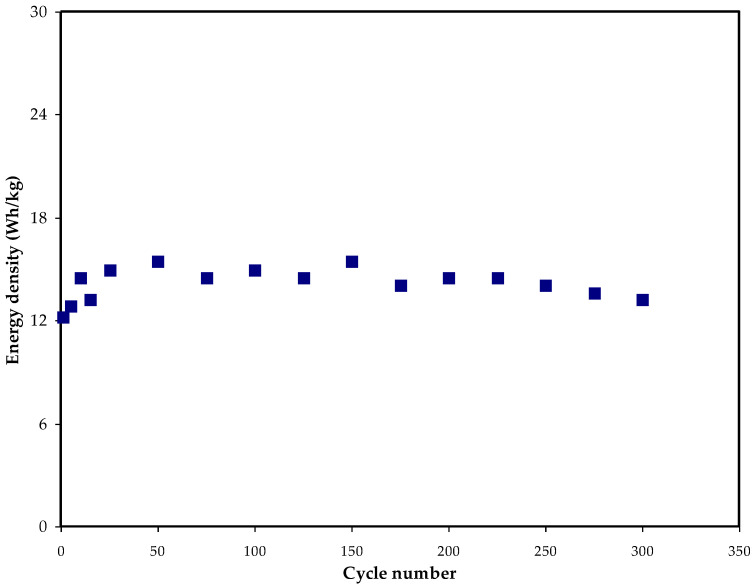
Energy density (*E_d_*) of the constructed EDLC against cycle number.

**Figure 9:**
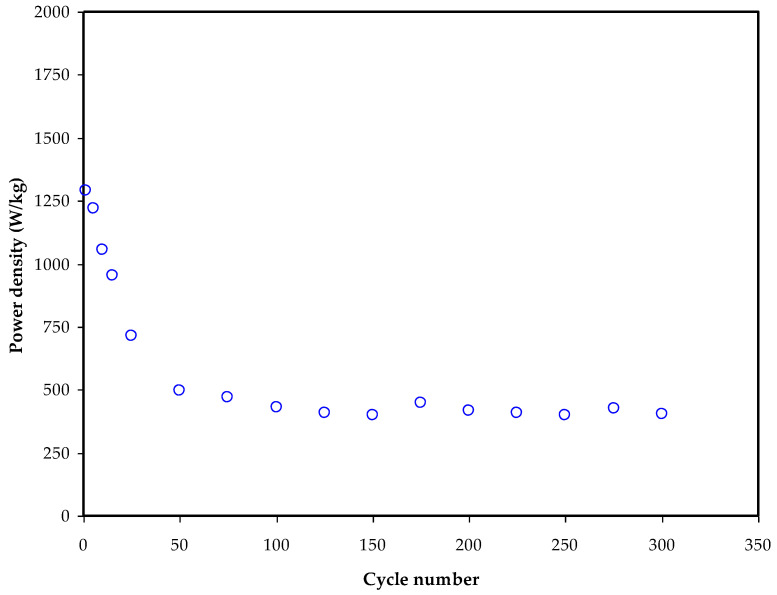
Power density (*P_d_*) of the constructed EDLC against cycle number.

Figure 8 shows that during the first cycle, *E_d_* is 12.2 Wh/kg. This climbs to around 16 Wh/kg after the 300th cycle and then stabilizes at about 15 Wh/kg on average. Consequently, we may deduce that ion conduction has a nearly identical energy barrier beyond the 50th cycle [71]. In earlier studies, the same *E_d_* value has been found for EDLCs made from a wide diversity of materials [72]. According to Hina and companions, EDLC lithium triflate (LiTf) content influences *E_d_*. For the carboxy methyl cellulose (CMC)–PVA–ammonium bromide (NH4Br) system, Mazuki and collaborators have shown an EDLC with *E_d_* of 1.19 Wh/kg [73]. The amount of energy that may be stored in EDLCs is greatly affected by ion collection at the electrode/electrolyte interface. Using a potato starch–MC–ammonium nitrate (NH_4_NO_3_)–glycerol system, Hamsan et al. [74] found an *E_d_* value of 2.25 Wh/kg. Prior to this study, we found that our prior work’s *E_d_* value of 0.77 Wh/kg was substantially smaller than that of the current work [75]. Plasticizer and Zn II metal complex may be responsible for the high *E_d_* value in this situation. This study has recorded higher *P_d_* compared to previous work without metal complex (MC:DN:NH_4_SCN) with *P_d_* of only 680 W Kg^−1^, while the *E_d_* is lower [31]. According to this study’s findings, biopolymer-based electrolytes are critical for energy storage applications. Figure 9 shows how the *P_d_* values may be determined if the *ESR* values are known. To begin with, the *P_d_* value is 1290 W/kg, but after 100 cycles, the *P_d_* value drops to 350 W/kg. The patterns of *ESR* and *P_d_* are quite similar. Electrolyte deterioration happens at higher cycle numbers when resistance develops owing to ionic buildup caused by the frequent charging and discharging [76]. The values of *E_d_* and *P_d_* in EDLCs are directly influenced by the mass loading of the active material. Improved electrochemical performance has been shown [77] as a result of the low current and small bulk loading.

## 4. Conclusions

A solution casting process was used to manufacture a polymer composite based on glycerol that plasticized the MC–DN–NH_4_SCN–Zn (II) complex. According to the electrochemical impedance spectroscopy (EIS), ionic conductivity with the addition of glycerol and Zn (II) metal complex is 3.5 × 10^−3^ S/cm. The values for the ion diffusion coefficient (D), carrier density (*n*), and ionic mobility (*µ)* have been determined to be 1.48 × 10^−6^ cm^2^/S, 3.4 × 10^20^ cm^2^/V. S, and 5.77 × 10^−5^ cm^−3^, respectively. The transference number measurement (TNM) analysis confirmed that ions are the predominant charge carrier, with an ionic transference (*t_ion_*) value of 0.972. The highly conducting sample was electrochemically stable until 2.05 V, indicating that the electrolyte was suitable for the EDLC device application, according to the linear sweep voltammetry (LSV) measurement. Over its full potential range, the cyclic voltammetry (CV) profile displayed no apparent redox peaks, indicating capacitance behavior. To assess the EDLC’s characteristics, the galvanostatic charge–discharge (GCD) curve was used for 300 cycles. Using the manufactured EDLC, the 1st cycle values of specific capacitance (*C_s_*), power density (*P_d_*), energy density (*E_d_*), and equivalent series resistance (*ESR*) were determined to be 105 F/g, 1290 W/kg, 12.2 Wh/kg, and ~65 Ω, respectively. The present study has shown a significant improvement in the properties of this biodegradable polymer electrolyte. The achieved outcomes are promising for the potential applications of this type of electrolyte in energy devices, including batteries, dye-sensitized solar cells, and hybrid supercapacitors. However, still more improvement is demanded to enhance the overall performance of this natural polymer electrolyte to become widely commercialized.

## Figures and Tables

**Table 1:** Different circuit element parameters of the produced composite polymer electrolyte.

Electrical Equivalent Circuit (EEC) Parameters	Values
Deviation from real axis (p_1_) (rad)	0.37
Reciprocal of capacitance (K_1_) (F^−1^)	1.2 × 10^4^
Constant phase elements (*CPE*1)	8.3 × 10^−5^

**Table 2:** Various ion transport values for the produced composite polymer electrolyte.

Ion Transport Parameters	Values
DC ionic conductivity ($\sigma_{DC}$) (S cm^−1^)	3.59 × 10^−3^
Diffusion coefficient (*D*) (cm^2^ s^−1^)	1.48 × 10^−6^
Ionic mobility (*µ*) (cm^2^ V^−1^ s^−1^)	5.77 × 10^−5^
Carrier density (*n*) (cm^−3^)	3.4 × 10^20^

**Table 3:** *C_s_* of the constructed EDLC utilizing glycerolized MC:DN:NH_4_SCN:Zn (II) metal complex electrolyte.

Scan Rate	Capacitance (F/g)
100	10.6
50	25.1
20	61.1
10	84.8

## Data Availability

Exclude this statement.
